# Hydrologically-driven crustal stresses and seismicity in the New Madrid Seismic Zone

**DOI:** 10.1038/s41467-017-01696-w

**Published:** 2017-12-15

**Authors:** Timothy J. Craig, Kristel Chanard, Eric Calais

**Affiliations:** 1 Institute of Geophysics and Tectonics, School of Earth and Environment, University of Leeds, Leeds, LS2 9JT UK; 2 Institute of Earth Sciences, University of Lausanne, Lausanne, Géopolis - CH-1015 Switzerland; 3 LASTIG LAREG, IGN, ENSG, Université Paris Diderot, Sorbonne Paris Cite, Paris Cedex 13,, 75205 France; 4 Ecole normale supérieure, Department of Geosciences, PSL Research University, 75231 Paris, France

## Abstract

The degree to which short-term non-tectonic processes, either natural and anthropogenic, influence the occurrence of earthquakes in active tectonic settings or ‘stable’ plate interiors, remains a subject of debate. Recent work in plate-boundary regions demonstrates the capacity for long-wavelength changes in continental water storage to produce observable surface deformation, induce crustal stresses and modulate seismicity rates. Here we show that a significant variation in the rate of microearthquakes in the intraplate New Madrid Seismic Zone at annual and multi-annual timescales coincides with hydrological loading in the upper Mississippi embayment. We demonstrate that this loading, which results in geodetically observed surface deformation, induces stresses within the lithosphere that, although of small amplitude, modulate the ongoing seismicity of the New Madrid region. Correspondence between surface deformation, hydrological loading and seismicity rates at both annual and multi-annual timescales indicates that seismicity variations are the direct result of elastic stresses induced by the water load.

## Abstract (web summary)

Large-scale changes in continental water storage have been shown to have an impact on seismicity. Here, the authors show that variation in the rate of microearthquakes in the New Madrid Seismic Zone coincides with hydrological loading in the Mississippi embayment at both annual and multi-annual timescales.

## Introduction

The lithosphere is constantly undergoing deformation as it adjusts to the redistribution of surface loads, particularly of continental hydrological origin^1–7^. This deformation takes place at timescales ranging from sub-annual to millennial depending on the causative mechanism, concerns spatial scales ranging from a few km to thousands of km, and produces geodetically observable deformation. Here, we focus on the effect that annual and multi-annual variations in continentally stored water mass have on lithospheric deformation, and how this influences seismicity in intraplate North America.

Despite the relatively small stress changes induced by annual-scale water load variations (typically less than a few kPa), the annual variation of terrestrial water mass has been suggested to modulate ongoing seismicity in a number of active tectonic environments, particularly in the Himalayas of Nepal^8–10^, California^11–13^ and beneath the Japanese Islands^14^, by varying either the stress state on active faults or pore-fluid pressures at earthquake nucleation depths. Microseismicity has also been proposed to be modulated by other comparable-magnitude stress variations, including atmospheric loading^15^ and oceanic and solid earth tides^16^. Non-volcanic tremor along the Cascadia subduction zone also correlates with a 0.1–1 kPa hydrological load variation^17^. In all of these environments, seismicity is the result of observable and ongoing tectonic processes, and the stressing rates due to water load variations are significantly smaller than the secular rates of stress accumulation, often by more than an order of magnitude^9^. Continental interiors present a different scenario, where secular stressing rates are unmeasurably low or negligible^18^. The influence of stress variations due to varying hydrological loads might therefore be expected to be greater in such settings, and yet, any modulating hydrological influence has been difficult to identify in intraplate earthquake sequences^19,20^.

**Fig. 1:**
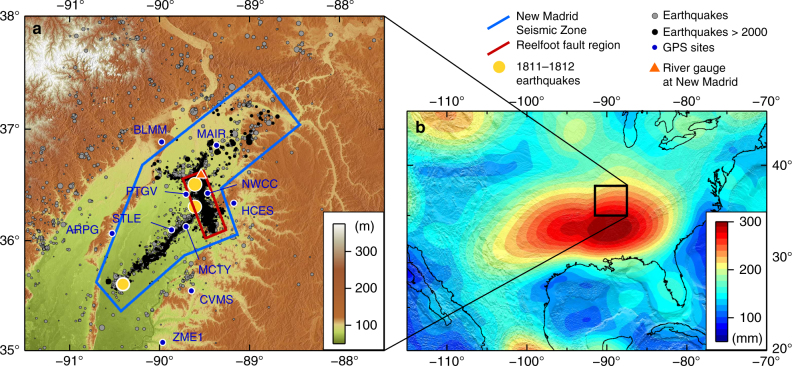
The New Madrid Seismic Zone (NMSZ). **a** Seismicity in the NMSZ from the full CERI catalogue^27^ (grey points), highlighting those since 1 January 2000 (black points). Yellow circles are the approximate locations of the 1811–1812 earthquakes. Earthquakes are scaled by magnitude. Blue and red boxes outline the approximate areas of the NMSZ and the Reelfoot fault, respectively. Other symbols indicate the river gauge at New Madrid (orange triangle), and continuously operating GPS sites in proximity to the NMSZ (blue circles). **b** Peak-to-peak variation in annual surface load from satellite gravity data, expressed as equivalent water height in millimetres

To address this, we focus on one of the type examples for ongoing natural intraplate seismicity in North America—the New Madrid Seismic Zone (NMSZ hereafter). While intraplate North America is tectonically quiescent, with limited seismic activity^21,22^ and little geodetically observable secular deformation outside the region affected by glacial isostatic adjustment^23,24^, the NMSZ (Fig. 1) in the central USA is a notable exception to this. The region experienced a pulse of four major (M > 7) earthquakes in 1811–1812^25,26^, and has been undergoing low-level seismic activity ever since^27,28^. Today, the NMSZ—one of the classic examples for continental intraplate seismic zones—continues to be one of the most seismically active regions of intraplate North America, although with earthquake magnitudes rarely exceeding M3. As a consequence, it is subject to some of the most concentrated seismological and geodetic instrumental monitoring of any intraplate region.

Secular stressing rates on NMSZ faults are unknown, but are likely to be very small given that two decades of modern satellite geodesy is yet to record any significant long-term strain accumulation^29,30^. As a result, the underlying causes of large earthquakes in the NMSZ—and in other similar intraplate settings—remain enigmatic. This paper does not address this issue but rather focuses on how annual and multi-annual stress changes of hydrological origin may affect the productivity of an ongoing seismicity cluster, regardless of its origin as a result of continuing secular strain or an ongoing aftershock sequence^31,32^. The magnitude of these hydrologically derived variations in stress is small compared with the long-term tectonic stresses, and, away from regions of major climate change (e.g., Greenland), or large-scale aquifer depletion (e.g., California), these variations take place around a long-term mean of zero. As a result, they can, at best, only modulate the seismicity, which must be driven at geological timescales by a different source of stress.

The NMSZ lies at the northern tip of the Mississippi Embayment (Fig.1a), and the fault system itself is intersected by the Mississippi River. Crucially, the NMSZ also lies on the northern flank of a major annually varying hydrological load associated with the variation of water contained in the lower reaches of the Mississippi catchment and along the Gulf Coast of the south-central USA (Fig. 1b), making it an ideal setting to study interactions between intraplate seismicity and hydrological loading.

By combining seismicity-rate observations from the local seismic network around the New Madrid Seismic Zone, geodetic observations of surface deformation and constraints on large-scale hydrological variations from both local river data and satellite gravity observations, we show that rates of microseismic activity (M < 2.3) in the NMSZ are modulated by long-wavelength variations in continental water storage, at both annual and multi-annual timescales. We demonstrate that this loading, which results in geodetically observed surface deformation on a centimetric scale, induces stresses within the lithosphere that, although of small amplitude (1–2 kPa), are capable of modulating the ongoing seismicity of the New Madrid region. The reduced water load in late summer and autumn promotes failure of the active fault system. Similarly, the lower water baselevel during 2005–2008 corresponds to a period of enhanced microseismicity. That this correspondence exists at two separate timescales, with no significant phase lag, suggests that the variation in seismicity rate is driven directly by changes in elastic stresses acting on the fault systems as a result of the changing hydrological load, and is not influenced by pore-fluid pressure variations.

## Results

### Temporal trends in seismicity

Instrumental monitoring of seismicity in the NMSZ has been in place since 1974, although the instrumentation and network distribution have evolved substantially over that period. Figure 1 summarises the regional seismic catalogue^27^ (see Methods for more details) from 1 January 2000 through 31 December 2015—a time period over which the network remains approximately uniform and stable. Present-day seismicity in the NMSZ is concentrated onto two principal structures: the SW-dipping Reelfoot thrust fault, and the right-lateral SW-striking Cottonwood Grove strike-slip fault, both of which are believed to have hosted events in the 1811–1812 sequence^32^ (Fig. 1a). Limited focal mechanism data for the region^22,33^ indicates that the ongoing seismicity is consistent with the regional tectonic stress state^34^. Of these two features, the Reelfoot fault is by far the more seismically active (2,559 of 3,277 earthquakes in the NMSZ region in the time period studied). Given that the response of differently orientated fault systems to regional loads will be different, we consider two regional sets of seismicity: one encompassing the whole of the NMSZ, and one focused specifically on a geographic region outlining the Reelfoot fault, with the aim of limiting the data set to earthquakes associated specifically with this thrust fault.

**Fig. 2:**
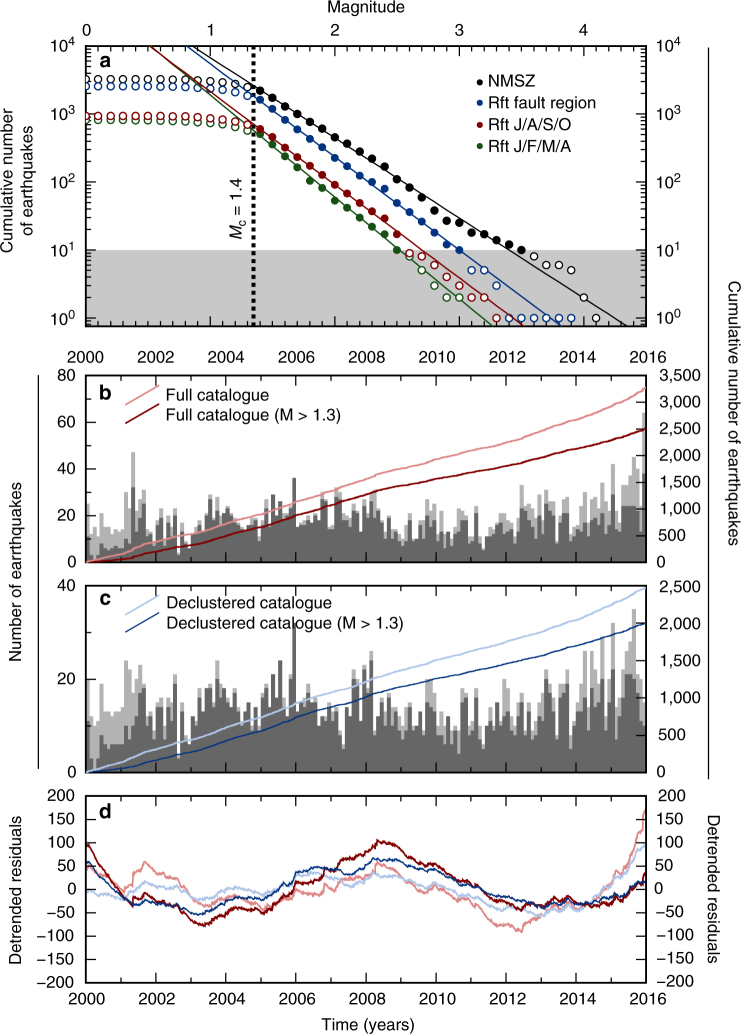
Seismicity in the New Madrid Seismic Zone (NMSZ). **a** Gutenberg–Richter plots for the seismic catalogue since 2000 shown in Fig. 1a, for the whole NMSZ (black points), for the Reelfoot fault region only (blue points), and for the Reelfoot fault region only in the months of January, February, March and April (green points) and in the months of July, August, September and October (red points). Filled circles are those used in calculating best-fit Gutenberg–Richter parameters, shown by the coloured lines. Unfilled circles are those excluded due to being either below the calculated magnitude of completeness, or when there are fewer than 10 recorded earthquakes. **b**, Histogram of earthquake frequency, binned at 0.1-year intervals for the full NMSZ catalogue (light grey), and considering only those earthquakes above the magnitude of completeness (dark grey). Lines indicate the cumulative number or earthquakes since 1 January 2000. **c** As in **b**, but for the NMSZ earthquake catalogue after declustering. **d** Cumulative number of earthquakes since 1 January 2000 with a best-fit linear trend removed. Colours are as in **b** and **c**

Assuming a Gutenberg–Richter magnitude–frequency distribution, this catalogue is complete down to, and including, M1.4 (see Methods), both across the whole NMSZ region, and in a region confined to the Reelfoot fault (Fig. 2a). As Fig.2a also demonstrates, this magnitude of completeness does not appear to vary significantly though the year, with M1.4 being the completeness magnitude for two separate 4-month periods (January/February/March/April and July/August/September/October, hereafter JFMA and JASO, respectively). To avoid biasing the temporal distribution of seismicity due to aftershock sequences, the catalogue is declustered (Fig. 2b, c; see Methods). This removes sharp jumps in the cumulative number of earthquakes following larger events, visible in the detrended accumulation rates (Fig. 2d), resulting in a smooth, although time-varying, seismicity rate.

**Fig. 3:**
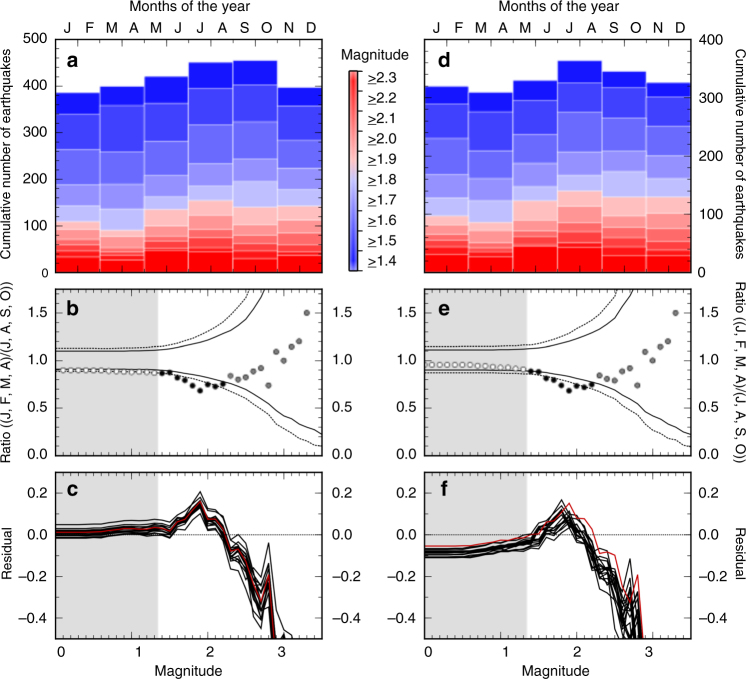
Analysis of seasonal trends in seismicity around the New Madrid Seismic Zone (NMSZ). **a** Histogram (in 2-month bins) for the number of earthquakes in the complete catalogue between 1 January 2000 and 31 December 2015 for the entire NMSZ region, as shown in Fig. 1a by the red box. Colours are indicative of the magnitude cut-off used in each case. **b** Ratio of the number of earthquakes occurring in the 4-month period encompassing January, February, March, April, to those occurring in July, August, September, October as a function of cut-off magnitude. Grey shaded areas indicate the magnitude of completeness. Dashed and solid black lines indicate the 99 and 95% confidence limits, respectively. Black points are those where the ratio exceeds the 95% confidence limit. **c** Results of a Jack-knife analysis of the seasonal trend. Lines indicate the residual at each magnitude band between the calculated ratio and the 95% confidence limit. Red line is for the full catalogue, as shown in **b**. Black lines are for the same catalogue with successive years of data removed. Confidence limits are estimated independently for each test. Grey area again indicates the magnitude of the completeness of the catalogue. **d**–**f** are as **a**–**c**, but for the seismicity catalogue after declustering

**Fig. 4:**
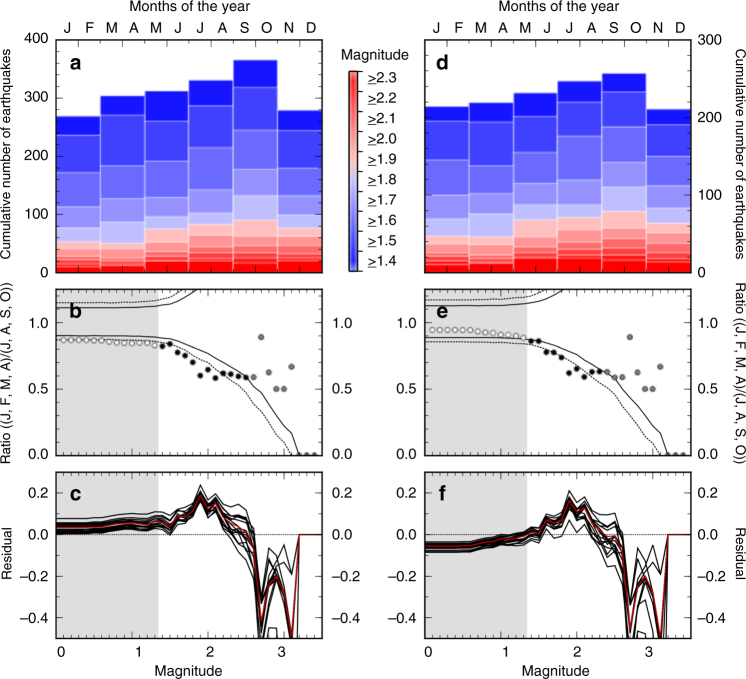
Analysis of seasonal trends in seismicity around the Reelfoot Fault. **a** Histogram (in 2-month bins) for the number of earthquakes in the complete catalogue between 1 January 2000 and 31 December 2015 for the area around the Reelfoot fault, as shown in Fig. 1a by the red box. Colours are indicative of the magnitude cut-off used in each case. **b** Ratio of the number of earthquakes occurring in the 4-month period encompassing January, February, March, April, to those occurring in July, August, September, October as a function of cut-off magnitude. Grey shaded areas indicate the magnitude of completeness. Dashed and solid black lines indicate the 99 and 95% confidence limits, respectively. Black points are those where the ratio exceeds the 95% confidence limit. **c** results of a Jack-knife analysis of the seasonal trend. Lines indicate the residual at each magnitude band between the calculated ratio and the 95% confidence limit. Red line is for the full catalogue, as shown in **b**. Black lines are for the same catalogue with successive years of data removed. Confidence limits are estimated independently for each test. Grey area again indicates the magnitude of the completeness of the catalogue. **d**–**f** are as **a**–**c**, but for the seismicity catalogue after declustering

In Fig. 3 (entire NMSZ) and Fig. 4 (Reelfoot fault region only), we present an assessment of the variability on an annual timescale of our two seismic catalogues (with and without declustering). In each case, the top panels show a set of histograms for the seismicity of the given region, stacked on an annual timescale, and divided into 2-month bins, and in all cases showing a tendency for high seismic activity in late summer/early autumn (JASO), and fewer earthquakes in spring (JFMA). This trend is much clearer for the Reelfoot catalogue (Fig. 4) than for the full NMSZ catalogue (Fig. 3), suggesting that the variation across the year is most apparent in earthquakes occurring on or close to the thrust-fault system, and that the other fault systems of the NMSZ are perhaps less susceptible.

Following Bollinger et al.^8^, we test this observation against the possibility of observing a similar ratio by chance. We take a synthetic data set consisting of 10,000 randomly generated seismicity catalogues with the same magnitude–frequency distribution as the observed earthquake catalogues, and calculate the 95 and 99% confidence limits for observing a ratio between events in JFMA to JASO that is significantly lower than 1 (Fig. 3b, e and Fig. 4b, e). At higher magnitudes, the number of earthquakes is insufficient to provide a robust, statistically significant trend. However, between M > 1.4 (the completeness magnitude) and M > 2.3 around the Reelfoot fault (or M > 2.2 for the full NMSZ catalogue; Fig. 3), the chance of observing such a large ratio between our two chosen time periods through random chance is <5%, and in both cases, <1% for specific magnitude bands. The ratio exceeds the confidence limits most at M > 1.9, where the variation results in approximately 60% more events in JASO than in JFMA.

Extreme climatic events are known to be capable of influencing seismicity^19,35^, and, despite declustering the catalogue, there is the potential that the apparent seasonal trend is influenced by a small number of extreme deviations, rather than an overall trend. To exclude this possibility, Figs. 3c, f and 4c, f show the results of a jack-knife analysis (see Methods), demonstrating that the exclusion of each calendar year of data separately from the data series does not result in any of the remaining catalogues not having a statistically significant seasonal variation between our JFMA and JASO periods, and the apparent seasonality is not the result of a single extreme annual excursion.

The applicability of a declustering routine to the New Madrid seismic catalogue remains open to question, due to the possibility that the observed seismicity is all part of an ongoing aftershock sequence. However, trends observed in the declustered catalogue are also present in those prior to declustering and indeed, as would be expected, using the full catalogue serves to emphasise the temporal trends at low magnitudes.

### Surface loading and induced deformation and stress

**Fig. 5:**
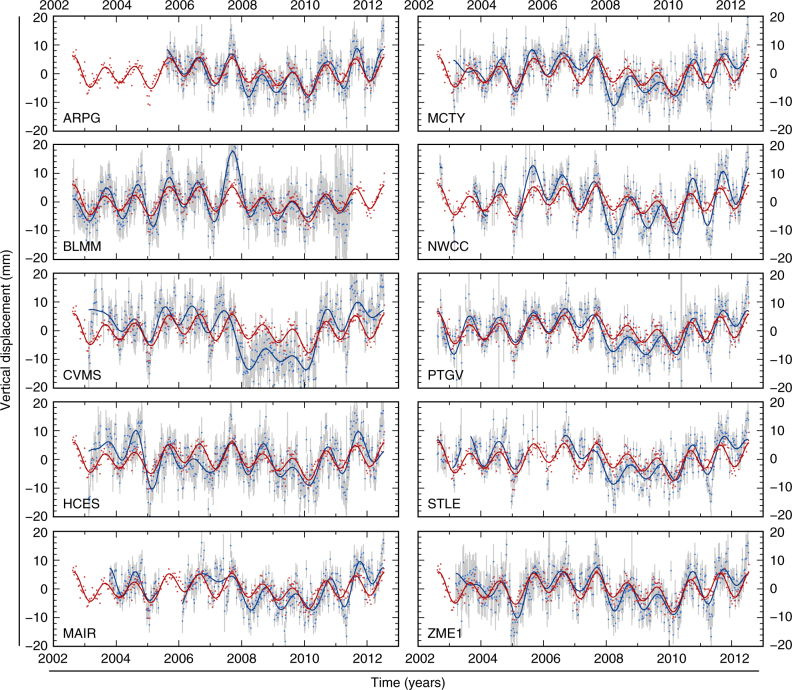
GPS displacement observations and gravity-derived displacement predictions. Vertical-component GPS displacements for 10 sites with the longest observation periods around the NMSZ (blue points). For processing details, see Methods. Site locations are given in Supplementary Table 1. Time series are corrected for instrumentation-related offsets. Grey bars are 1*σ* uncertainties. GPS site locations, identified by the site ID shown in the lower left of each panel are shown in Fig. 1a. Red points are vertical-component displacements calculated for a layered-elastic spherical Earth response to GRACE-observed surface load variations (see Methods). A best-fit linear trend has been removed from both GPS and gravity-derived time series. Blue and red lines show a best-fit 24-component Fourier series to the GPS and gravity time series, respectively

In addition to seismological monitoring, the NMSZ is also heavily instrumented with continuous Global Positioning System (cGPS) installations (blue dots, Fig. 1a). Estimates of secular strain accumulation in the NMSZ from geodetic observation, once controversial^36,37^, now show insignificant (<3 × 10^−9^ yr^−1^) secular strain accumulation over the last two decades^29,30^. However, cGPS position time series do show a strong annual variation in surface displacements, most notable in the vertical component, with peak-to-peak amplitudes of ~12 mm (Fig. 5). As with other regions^3–5,38,39^, this is believed to be linked to the annual climatic cycle, and, in particular, to the redistribution of continental water masses in the Mississippi basin over the year.

Satellite gravity measurements from the Gravity Recovery and Climate Experiment (GRACE) are now commonly used to quantify spatial and temporal variations in the distribution of continental water masses. In addition to detecting longer-term trends related to climatic variations^40,41^, GRACE observations show a strong annual signal in regions of large hydrological load change^39^. This is the case along the lower Mississippi and Gulf Coast regions of the USA, extending up to the NMSZ (Fig. 1b). Although these observations are limited to a minimum spatial resolution of ~200–300 km, recent work has shown that these observations are capable of explaining the majority of the non-linear signal seen in continental cGPS sites at both regional^3–5,9^ and global scale^42^. Whereas cGPS installations provide high accuracy, high temporal resolution displacement observations at discrete points on the Earth’s surface, observations from GRACE have the advantage that they are spatially continuous and uniform, and so allow us to calculate the full displacement, strain and stress fields throughout the Earth for the solid-Earth response to variations in continental water mass, using a spherical layered elastic Earth model^42^ (see Methods). The calculation for the solid-Earth response to the non-linear load derived from variations in the Earths gravity field can be validated by comparison to the observed global geodetic surface displacements, and then used to evaluate stress changes on the NMSZ faults that result from the redistribution of continental water masses.

While the GRACE observations we use incorporate atmospheric and oceanic loading components (coherent with GPS position time series used), the amplitude of the load due to these sources in the NMSZ is small compared to that due to variation in continental water mass^43^.

The comparison between the observed vertical displacements at high-quality cGPS sites around the NMSZ (blue points, Fig. 1a) and the predicted vertical displacements induced by GRACE-observed surface-loading variations shows a good match, which demonstrates that the seasonal predictions appropriately predict both the amplitude and phase of the load-induced surface displacements (Fig. 5).

### Hydrologically influenced surface deformation and seismicity

**Fig. 6:**
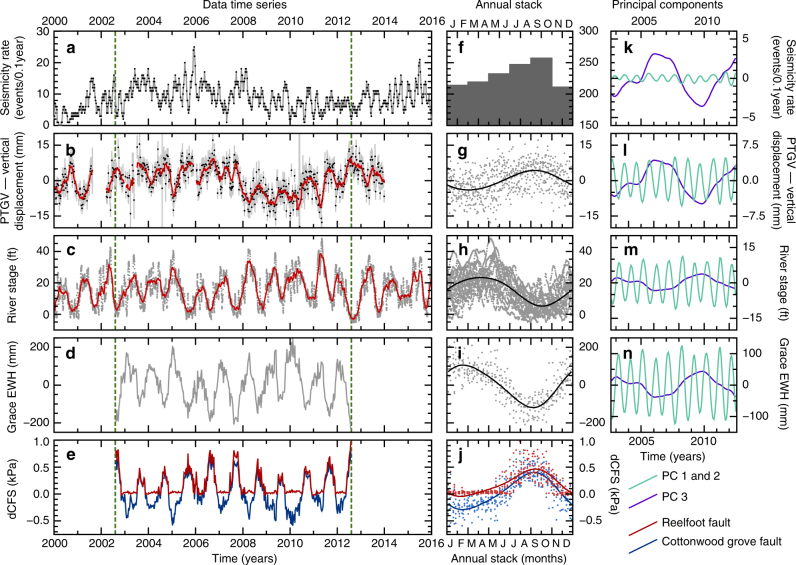
Data time series annual stacks and M-SSA analysis. **a** Seismicity rate for the Reelfoot fault region (Fig. 1). **b** Vertical-component GPS displacements from site PTGV (black points), with 0.2-year running average (red). See Fig. 1a for site location. **c** River stage observations from New Madrid (orange triangle, Fig. 1a) with 0.2-year running average (red). **d** GRACE gravity observations for the New Madrid region. **e** Calculated Coulomb failure stress variations from GRACE loading variations for the Reelfoot fault (red) and Cottonwood grove fault (blue). **f** Earthquake frequency histogram, stacked on an annual timescale (as in Fig. 3d). **g**–**j**, as in **b**–**e**, but stacked on an annual timescale. Curves are a best-fit four-component annual Fourier series to the stacked data. **k**–**n** The first three principal components for seismicity rate, GPS displacement, river stage and GRACE gravity, respectively, as determined from Multi-channel Singular Spectrum Analysis (see Methods) on the time period common to all data time series (green dashed lines, **a**–**e**). PCs 1 and 2 are both approximately annual, and are shown combined. PC 3 is a longer-term multi-annual signal common to all four data time series Ed: Please ask the authors to edit the title of figure 6 to remove.

In Fig. 6, we investigate the relationship between continental water storage, surface deformation and seismicity. We combine time series for the seismicity rate in the Reelfoot region, vertical-component GPS displacements at site PTGV, Mississippi river stage data from a river gauge at New Madrid, GRACE-observed loading and calculated stress changes induced by the GRACE loads on the principal faults, and assess the presence of common temporal modes in these data. A strong correlation is observed between all time series, with the correspondence between GRACE loading and Mississippi river stage confirming the hydrological origin of the loading signal. We also see a good correlation between the peak in seismicity rate, and the peak in the predicted stress amplitudes on the fault system, as derived from GRACE data. This demonstrates that despite the long wavelength of the GRACE observation data, it is sufficient to image the causative load changes capable of driving the variation in seismicity on an individual fault system. Annual stacks reveal a strong correlation between GPS displacement and seismicity, with a peak in JASO and a trough in JFMA, and an anti-correlation between the seismicity, and river stage and GRACE loading, with minimum load corresponding to maximum seismicity.

To probe beyond the initially identified annual signal, we apply Multi-channel Singular Spectrum Analysis (M-SSA)^44,45^ to the four observational datasets (seismicity rate, vertical-component displacement at PTGV, river stage, GRACE equivalent water height; see Methods) for the 10-year period (2002.6–2012.6) where they overlap. This form of principal component analysis simultaneously takes advantage of the spatial and temporal correlations in time series to extract empirical orthogonal basis functions that represent their common modes of spatiotemporal variability. A significant benefit of this method is to allow extracting oscillations and non-linear trends without a priori knowledge about their period and amplitude or their spatiotemporal structures^46^. Figure 6k–n shows the three most significant principal components from the four data series. Component pair 1 and 2 represents an oscillation of approximately annual period, and, as with the annual stacks in Fig. 6f–i, show a strong (anti-)correlation between the seismicity, surface displacement, GRACE load and river stage. In addition, we identify a third component showing a multi-annual, non-linear trend common to all four data series. Once detected by M-SSA, this multi-annual component can be identified visually on the actual time series (Fig. 6a–d), in particular in the seismicity rate and GPS displacement. This multi-annual signal shows the same (anti-)correlation between the seismicity, surface displacement, GRACE load and river stage as the annual one. It therefore appears that both an annual and multi-annual hydrological signal are present, and have an impact on surface displacements and seismicity rates.

**Fig. 7:**
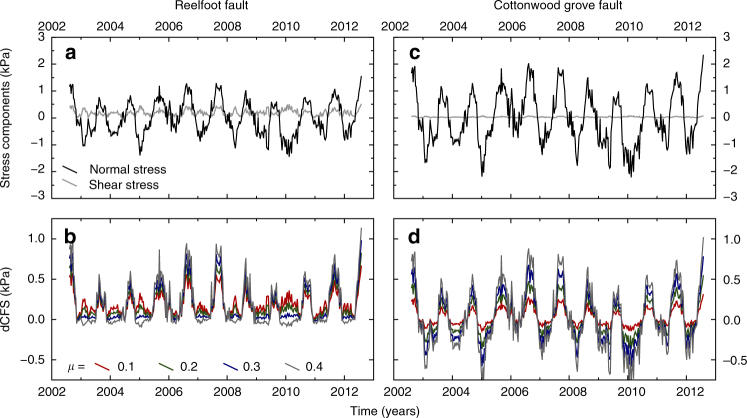
Stress variations for the two fault systems. The top two panels (**a**, **c**) show variations in shear (grey) and normal (black) stress resolved on to the fault plane. The lower two panels (**b**, **d**) show changes in Coulomb stress calculated from the normal and shear stresses above, using various coefficients of friction from 0.1 to 0.4. The plot shown in Fig. 6 assumes a coefficient of friction of 0.4. The left two panels show values calculated for the Reelfoot thrust fault, and the right two panels show the same values calculated for the Cottonwood grove strike-slip system

From the GRACE-derived deformation field used to match the surface deformation shown in Fig. 5, and using seismological constraints on the geometry of the principal faults of the NMSZ^30,47–49^, we resolve the induced full stress tensors that result from the migratory surface loading onto the fault planes of the Reelfoot thrust fault and Cottonwood grove strike-slip fault, and express these as variation in the Coulomb failure stress (dCFS hereafter; Fig. 6e, j; see Methods). For the Reelfoot fault, we calculate a peak-to-peak variation of ~1 kPa over each yearly cycle, with the annual average peak occurring in September, indicating that even small magnitude stress changes are sufficient to influence the seismicity rate in this area. In contrast to our observation that the annual variation in seismicity is most clearly observed when the seismic catalogue is restricted to the Reelfoot fault region (Fig. 4), the predicted amplitude of the stress variation is similar for the Cottonwood grove fault (Fig. 7), perhaps suggesting that we are limited by much lower seismic activity along this fault in observing any seasonal trend.

Variations in the geometry of the Reelfoot and the Cottonwood grove faults introduce some uncertainties in the dCFS calculations carried out here. The Cottonwood grove fault is very linear with a simple planar geometry, while the orientation of the Reelfoot fault may vary slightly across the intersection with the northern end of the Cottonwood grove fault^48,49^. The calculations shown on Figs. 6 and 7 use the fault geometries of Boyd et al. ^30^ and are accurate for the well-defined northern section of the Reelfoot fault. The magnitude of the calculated stresses on each fault segment will vary slightly within the range of estimated fault orientations, but the phase, more critical for our interpretation, is dominated by the spatial migration of the load around the receiver fault and is insensitive to minor changes in the geometry of the receiver fault. The effect on the magnitude of the stress variation is, however, small when compared to other uncertainties in the elastic structure and, for dCFS, the effective coefficient of friction (Fig. 6b, d).

The annual minimum in calculated dCFS for the Reelfoot fault does not vary significantly over the timespan of this study. However, the peak value within each annual cycle does vary, by up to a factor of ~2. The timescale and phase of this variation matches with that of the multi-annual signal extracted from the M-SSA analysis. The period of maximum dCFS peaks (mid-2005–mid-2009) results from a period of high-amplitude troughs in GRACE load, and matches with a series of major lows in river stage, indicating that this multi-annual signal also has a hydrological origin.

In contrast to recent findings in northern California^13^, where variations in microseismicity rates along strike-slip faults around the Central Valley appears to correspond to variations in hydrologically induced shear stress, the dominant controlling mechanism on the Reelfoot fault appears to be variations in the normal stress (Fig. 7a, c). In the case of the Reelfoot fault, even with very low effective coefficients of friction on the fault, the seasonal signal is dominated by the annual variation in the normal stress, with minimal variation in the shear stress.

### Fault mechanics and the mechanism of hydrological forcing

Two principal mechanisms have been suggested by which hydrological influences can impact on earthquake occurrence: variations in pore-fluid pressure at hypocentral depths, and direct stress effects on the fault plane. In the latter case, there should be little or no time delay between hydrological indicators and seismicity rate. In the former, seismicity rate variations will be delayed relative to hydrological variations by a time lag dependent on the depth of earthquake nucleation and the hydraulic diffusivity of the material between the surface and the earthquake hypocentre. Previous examples of hydrologically modulated seismicity have either been conclusively shown to be shallow seismicity modulated by pore-fluid pressure changes in the upper few kilometres of the crust^50,51^, or are ambiguous as to the cause of modulation, due to the presence of only a single (annual) frequency of hydrological loading, with either a ~0-month phase lag for a direct stress effect, or a ~6-month phase lag for a pore-fluid effect^8,9,11,52^.

In the case of New Madrid, there is a clear inverse correlation between GRACE-observed loading and both seismicity and surface displacement at both an annual period and at a multi-annual timescale (Fig. 6). Not only is this consistent with our calculations for the stresses exerted on the fault systems by the hydrological load (Fig. 6), but the existence of an inverse correlation at multiple time periods allows us to rule out a pore-fluid pressure-related effect. Any time lag due to hydraulic diffusion for pore-fluid pressure waves through the crust should be consistent at all loading periods. At an annual period, any fluid-related effects would have to be operating with a phase lag of ~6 months to explain the variation in seismicity, and while this would produce plausible crustal hydraulic diffusivity values^19,50,51^, it is not consistent with the longer-period inverse correlation between seismicity and loading, which would require a second, and different, range of hydraulic diffusivity values.

The observed variation in seismicity rates in the NMSZ appears to be approximately in phase (to within <3 months) with the stresses resulting from variation in surface loading at annual and longer periods. This contrasts with the results of Bettinelli et al. ^9^, for Nepal, where there is an observed phase lag between the predicted stress and seismicity variations, with the correlation instead appearing to be between seismicity and stress rate.

Following Ader et al. ^53^, for a rate-and-state fault subjected to a periodic variation in shear-stress, we expect the amplitude (*β*) and phase (*φ*) of the change in seismicity rates to depend on the relationship between the period of the stress variation (*T*) and a critical period (*T*_c_), such that $T_{\rm c} = 2\pi a\sigma (\dot \tau _{\rm r})^{ - 1}$, where *a* is a rate-and-state fault parameter, *σ* is the effective normal stress and $\dot \tau _{\rm r}$ is the secular stressing rate. When *T* < *T*_c_, $\varphi \approx 0$, whereas at *T* ≥ *T*_c_, $\varphi \approx \frac{\pi }{2}$. *β* peaks at $T \approx T_{\rm c}$, and decays away as $T \to 0,\infty$.

In the case of Nepal, *T*_c_ ≈ 1 year, leading to a high-amplitude response to the stress rate $\left( {\varphi \approx \frac{\pi }{2}} \right)$, rather than the stress, and negligible response to much shorter period (but higher stress amplitude) tidal stresses^9,53^. For the NMSZ, we suggest that the much slower secular stressing rate $\left( {\dot \tau _{\rm r}} \right)$ instead leads to *T*_c_ ≳ 10 years, such that the system is sensitive to loading at both 1 and ~ 8-year periods. Figure 6k indicates that the seismicity response is more sensitive to loading at multi-annual than at annual periods, matching with a decay in *β* as *T*/*T*_c_ decreases, provided that *T*_c_ is greater than the longest observed period. Unlike in the Nepali case, if $\frac{T}{{T_c}} < 1$ at our loading periods, we expect $\varphi \approx 0$, as seen in the data at both annual and multi-annual loading periods. That the critical time is much greater in the slowly-deforming plate interior setting of the NMSZ than it is at an active plate boundary, suggests that seismicity rates in such settings are more sensitive to periodic stress variations, especially at longer multi-annual periods, than their more active counterparts.

## Methods

### Seismicity analysis

We use the seismic catalogue for the New Madrid region maintained by the Center for Earthquake Research and Information (CERI) at the University of Memphis^27^. While this catalogue extends back to 1974, we limit our data set to a sub-catalogue extending from the 1 January 2000 through to 31 December 2015, in order to maximise the uniformity of the catalogue, and to limit our susceptibility to any biasing due to changes in instrumentation or data quality. Over this monitoring period, the stations distribution and operation have remained reasonably stable (see Supplementary Fig. 1), and so should not lead to any major changes in the detection sensitivity or biasing in the catalogue due to network evolution.

The complete CERI earthquake catalogue is declustered prior to regional selection using the routines of Reasenberg^54^. This method aims to remove earthquake sequences where earthquakes form a chain connected in space and time, leaving only the initial earthquake in a given sequence. We use the same parameters determined in the original study, calibrated using the seismicity of Southern California, adapted for the spatial resolution in event location of the CERI catalogue. We note that the argument can be made that a truly successful declustering of the New Madrid catalogue might lead to the removal of all events back to the major earthquakes in 1811–1812, under the interpretation that all ongoing earthquakes are aftershocks of these events. However, our application of a declustering routine is instead aimed at removing the rapid pulses in seismic activity related to the small-scale aftershock clouds from discrete events within our observation period. The influence of these events can been seen particularly on the detrended cumulative frequency plots shown in Fig.2d, where they appear in the catalogue prior to declustering (red lines) as sharp jumps in the cumulative number of earthquakes. In the declustered catalogue (blue lines), these sharp jumps have been successfully removed by our declustering approach.

Magnitude of completeness and Gutenberg–Richter values, as shown in Fig.2a, are determined using a maximum-likelihood estimator. Cumulative magnitude bands with fewer than 10 earthquakes were excluded from this assessment, to minimise the effect of small-population values in biasing the fit.

Ratios shown in Figs. 3 and 4 are calculated by dividing the number of earthquake above a given magnitude threshold in the 4-month period JFMA by the number occurring in the 4-month period JASO. The confidence intervals shown are calculated based on applying the same process to 10,000 randomly generated seismicity catalogues with the same magnitude–frequency distribution, and taking envelopes that encompass the maximum (or minimum) 9,500 and 9,900 of the resultant ratios in each magnitude band for the 95 and 99% limits, respectively^8^.

The jack-knife analyses shown in Figs. 3c, f and 4c, f are determined by repeating the ratio-based analysis of Figs.3b, e and 4b, e, but removing one calendar year of data from the seismic catalogue in each test, after declustering (if used) and regional selection have been applied. The residuals shown in Figs. 3c, f and 4c, f are then determined by differencing the observed ratio and the 95% confidence interval for each test, such that positive values indicate datasets and magnitude bands where the confidence interval has been exceeded. Full details of each individual jack-knife are shown in Supplementary Figs. 2–5.

### GPS data

We use GPS position time series from the Central and Eastern US-focused study of Craig and Calais^29^, where the full processing routine is described in detail. GPS data, most critically those of the Mid-America GPS Network^55^, are publicly available from the UNAVCO and CORS archives (http://www.unavco.org/ and http://www.geodesy.noaa.gov/CORS/, respectively), and were processed using the GAMIT–GLOBK software package^56^. Time series shown in Figs. 5 and 6 (and Supplementary Fig. 6) are weekly position solutions determined from the combination of seven daily position solutions within each GPS week, initially tied to the International Terrestrial Reference Frame (ITRF-2008)^57^. The data shown have been subjected to the removal of offsets associated with instrumentation changes (by allowing for a one-off three-component offset at the time of instrumentation change) and then to a linear detrend.

For this study, we select the 10 GPS sites around the NMSZ with the longest overlap with the period for which we also have gravity data. Compared to the site selection in Craig and Calais^29^, this excludes the site RLAP, which, while it operated throughout our study period, underwent a technical fault for a period of ~4 years (from late 2005 through mid-2009), leading to an incomplete overlap with the gravity data, with large time gaps. A further 15 sites across the wider central USA are shown in Supplementary Fig. 6. Site locations, data completeness and the amplitude of the annual signal, are given in Supplementary Table 1.

### Gravity data processing

We use the global mass redistribution at the Earth surface estimated from gravity measurements by the satellite mission GRACE (Tapley et al., 2004) [38] from 2002 to 2012. We use 10-day Level-2 solutions produced by the CNES/GRGS^58^, to which we add back the atmospheric and non-tidal oceanic loading contribution^43^. Solutions are expressed in terms of Stokes’ coefficients representing the gravitational effects of non-modelled phenomena (continental water, sediments displaced, vegetation, oceanic and atmospheric mass variations, etc.), then converted into geoid and water mass coefficients (mm of equivalent water height) by isotropic filtering^40^. We interpolate 10-day 1-by-1 degree grids of water mass from the water mass coefficients and removed a time average from each 10-day solution so that the solutions are expressed with respect to the mean solution over the time span of analysis. We account for the geocentre motion (motion of the centre of mass of the Earth system with respect to the centre of figure of the solid Earth) and associated deformation field induced by the non-observable degree-1 loads from a comparison between our model and GNSS observations at a globally distributed network of sites^42^.

### Modelling the solid Earth response to surface loads

We compute surface displacements induced by variations of surface loading using a numerical model based on a spherical harmonics decomposition of the GRACE-derived loads that uses the Love numbers theory. We compute surface displacements induced by a unit load for each spherical harmonic of the decomposition of the load by solving a system of equations for the elastic deformation of a self-gravitating spheroid body, similar to classical normal mode theory, as used in seismology. We then combine displacements for each spherical harmonic to obtain surface displacements induced by the global surface loading at a specific time, longitude and latitude.

We use a modified PREM^59^, in which the oceanic crust is replaced by a continental one (CRUST 2.0) to compute load Love numbers and Green’s functions for horizontal and vertical displacements caused by unit, harmonic loading functions. We then convolve the Green’s functions with the spatially and temporally varying surface load derived from GRACE from 2002 to 2012 (the period from 2012 to 2016 contains large gaps in the gravity data time series), to compute model surface displacements at the location of the set of cGPS stations in the NM region.

We also compute the full time-varying stress tensors on the Reelfoot and Cottonwood grove faults, assuming their geometry^30,47,48^, at a 20 km depth, from the three-dimensional full load derived from GRACE. Note that considering the wavelength of the load, calculated stresses will not vary significantly within the thickness of the crust. We then quantify the fault susceptibility to failure under annual surface loading, using Coulomb failure assumptions. The Coulomb failure stress is given by: $\sigma _{\rm c} = \left| \tau \right| + \mu (\sigma _{\rm n} - p) + C$, where $\tau$ is the shear stress on the fault (along strike $\tau _{\rm s}$ and dip $\tau _{\rm d}$), $\sigma _n$ is the normal stress on the fault, *μ* is the friction coefficient, *C* the cohesion and *p* the pore-fluid pressure. Assuming that *p*,* C* and *μ* are constant in time, the change of Coulomb stress is given by ${\mathrm{\Delta }}\sigma _{\rm c} = {\mathrm{\Delta }}\left| \tau \right| + \mu \sigma _{\rm n}$. Note that by convention ${\mathrm{\Delta }}\sigma _{\rm c}$ is positive in tension. Accordingly, a Coulomb stress increase should enhance seismicity. In Fig. 7, we show separate shear and normal stress variations for the two fault systems, along with the effect of changing the value of *μ* used in calculating the Coulomb stress change. For the calculations shown in Fig.6, we use *μ* = 0.4, but changing this value largely affects the amplitude of the stress variations, rather than changing the features of the time series.

### Multichannel Singular-Spectrum Analysis

M-SSA exploits the covariance information contained in a series of lagged copies of all timeseries over a sliding M-point window^44,45^. The method starts by forming the matrix that includes M time-delayed copies of the original time series. It then computes the covariance matrices between all pairs of time series, which are then used as blocks of a grand covariance matrix that contains both spatial and temporal correlations. This latter matrix is used to calculate eigenvectors to spatiotemporal empirical orthogonal functions (ST-EOFs). Each eigenvalue carries a given amount of variance from the overall data set. In practice, M-SSA is a principal component analysis performed jointly in space and time. Eigenvalues that form pairs with corresponding ST-EOF in phase quadrature (such as 1 and 2 in Fig. 5) indicate the presence of oscillatory modes. Such pairs of ST-EOFs can be seen as data-adaptive counterparts of the sine and cosine functions in the usual Fourier analysis of time series.

Here we used M=400 days in order to capture the annual signal included tin the time series. We use the time of GRACE observations (one every 10 days) as our basis time vector and resample the GPS, river stage height, and seismicity and accordingly. We run a 3-epoch moving average through the time series in order to filter out some of the high-frequency noise. We use a single-channel SSA, i.e., M-SSA performed only for each time series independently, to fill the small gaps observed in some of the time series^46^. We finally run the M-SSA on the normalised, corrected, time series.

### Data availability

All data used in this study are publicly available. The seismic catalogue used in this study is maintained by the Centre for Earthquake Research and Information at the University of Memphis, Tennessee, and can be accessed at http://www.memphis.edu/ceri/seismic/catalog.php. GPS data are available though UNAVCO (http://www.unavco.org/) and CORS (http://www.geodesy.noaa.gov/CORS/) Data Archives. Gravity data are available through the Groupe de Recherche en Géodésie Spatiale, France, at http://grgs.obs-mip.fr. River stage data are available from the US Army Corps of Engineers, online for initial data (http://rivergages.mvr.usace.army.mil), and upon request from USACE for quality-controlled data. All river data remain preliminary and are subject to change.

## Electronic supplementary material

Supplementary Information
