# Supplementary material for: Hydrologically-driven crustal stresses and seismicity in the New Madrid Seismic Zone
Source: Nat Commun. 2017 Dec 15;8:2143. doi: 10.1038/s41467-017-01696-w (PMC5732252; doi:10.1038/s41467-017-01696-w)
Supplement: Supplementary file 1 — Supplementary Information [file 41467_2017_1696_MOESM1_ESM.pdf]

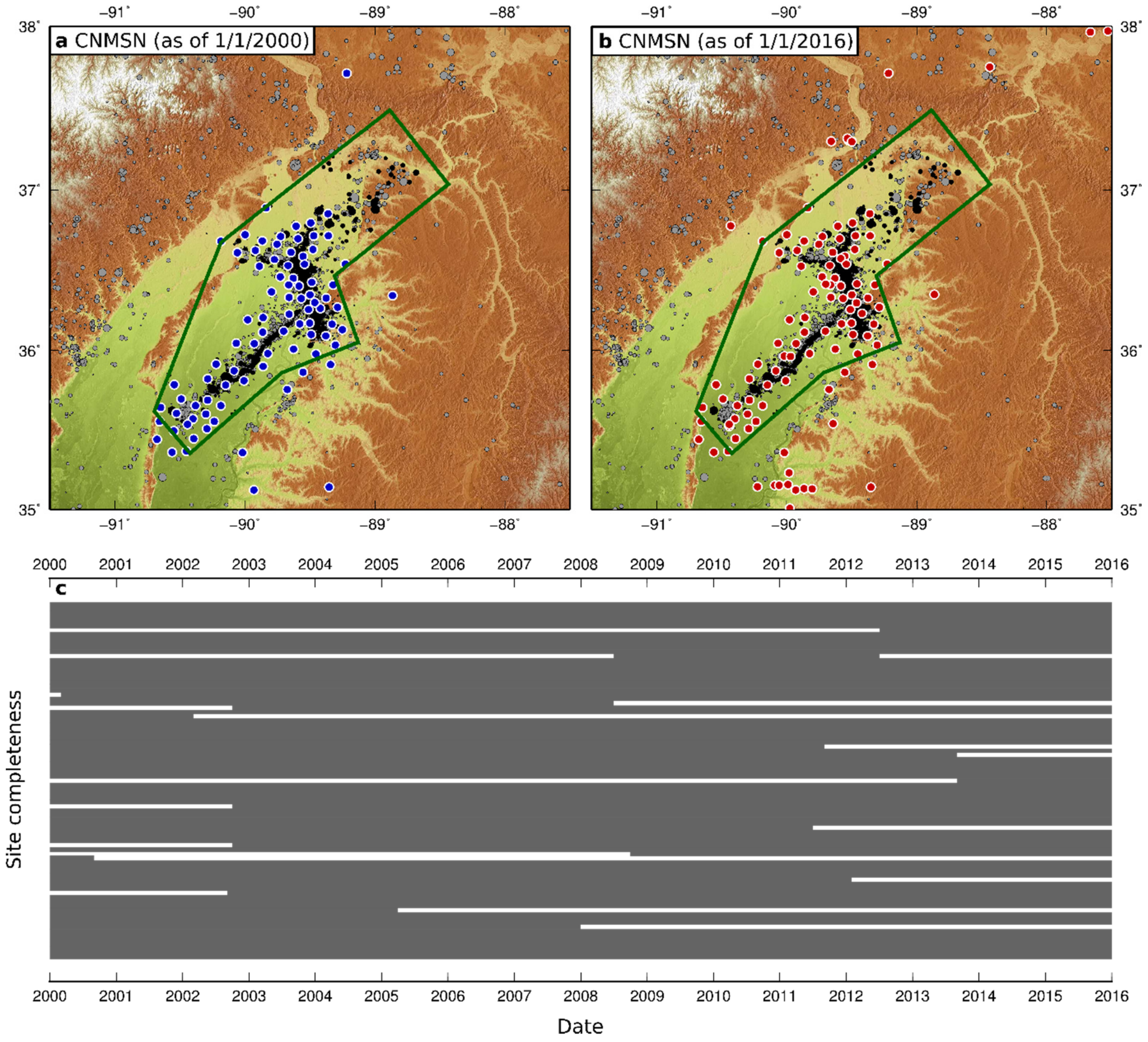

Supplementary Figure 1 | New Madrid seismic network stability. **a**, Seismic stations operating within the Cooperative New Madrid Seismic Network as of 1st January 2000. **b**, Seismic stations in the Cooperative New Madrid Seismic Network operating on 1st January 2016. **c**, Seismic station operating history for all sites within the New Madrid region between 2000 and 2016. Operational epoch is based on data holdings available at the Incorporated Research Institutions for Seismology Data Management Center (http://ds.iris.edu/mda/NM). Each grey bar indicates the operating period of a single seismic station shown on panels **a** and **b**.

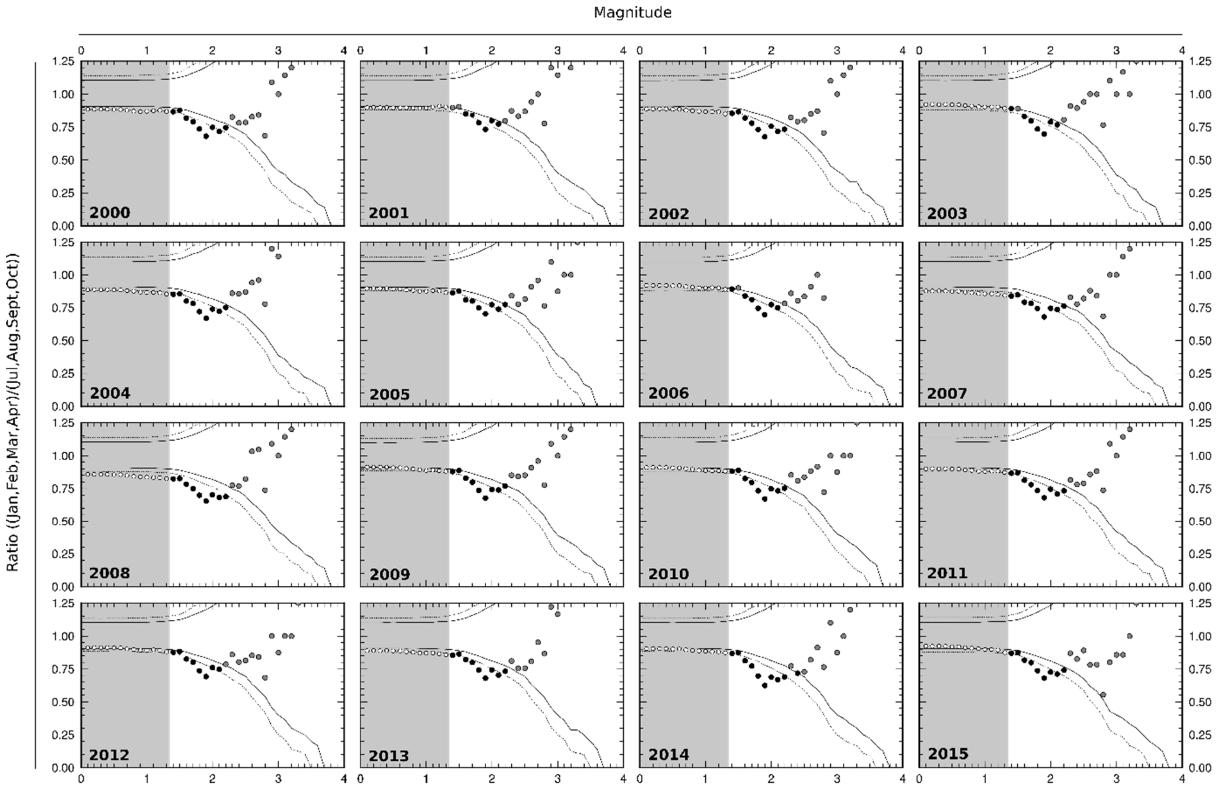

Supplementary Figure 2 | Full jack-knife analysis for complete seismic catalogue (New Madrid Seismic Zone). Ratio of the number of earthquakes occurring in the four-month period encompassing January, February, March, April, to those occurring in July, August, September, October as a function of cut-off magnitude for the full seismic catalogue in the New Madrid region (blue box, Figure 1a). Grey shaded areas indicate the magnitude of completeness. Light and dark blue areas indicate the 99% and 95% confidence limit respectively. Black points are those where the ratio exceeds the 95% confidence limit. Number in the lower left corner of each panel indicates the year of data removed from the 1/1/2000 to 31/12/2015 overall time period.

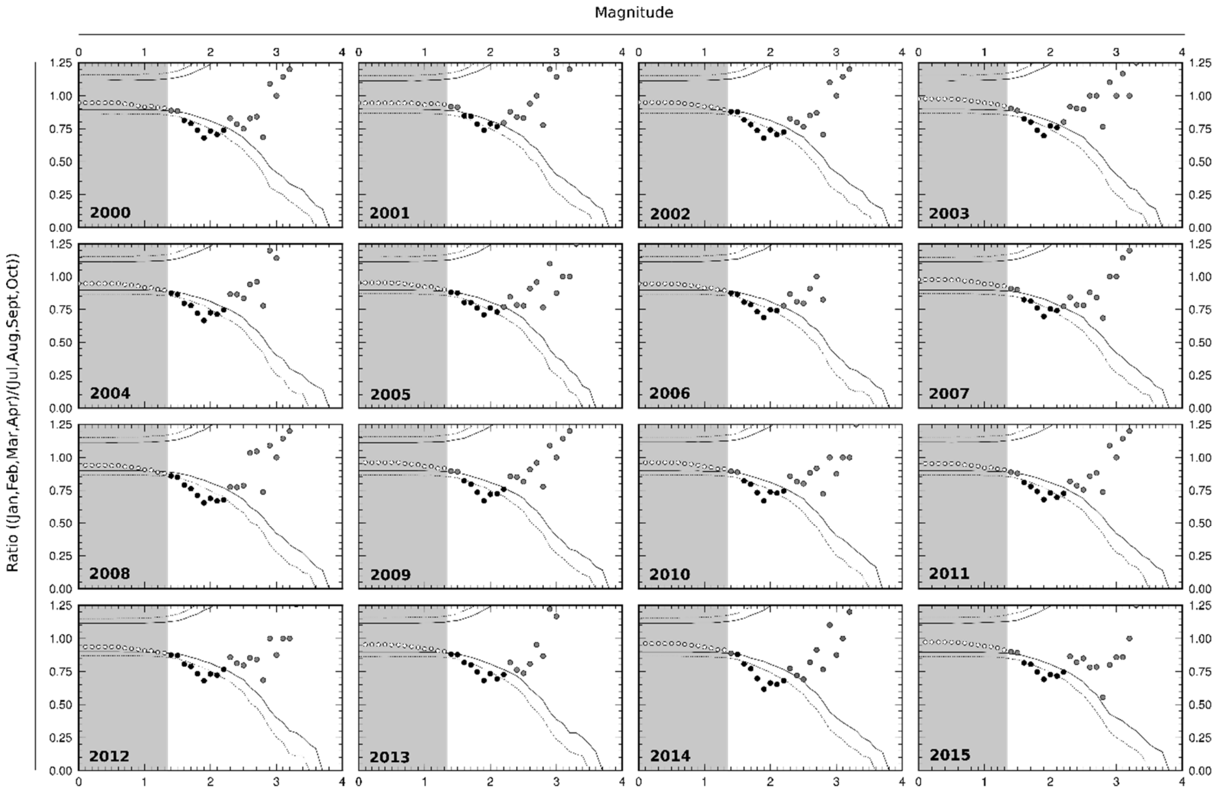

Supplementary Figure 3 | Full jack-knife analysis for declustered seismic catalogue (New Madrid Seismic Zone). Ratio of the number of earthquakes occurring in the four-month period encompassing January, February, March, April, to those occurring in July, August, September, October as a function of cut-off magnitude for the declustered seismic catalogue in the New Madrid region (blue box, Figure 1a). Grey shaded areas indicate the magnitude of completeness. Light and dark blue areas indicate the 99% and 95% confidence limit respectively. Black points are those where the ratio exceeds the 95% confidence limit. Number in the lower left corner of each panel indicates the year of data removed from the 1/1/2000 to 31/12/2015 overall time period.

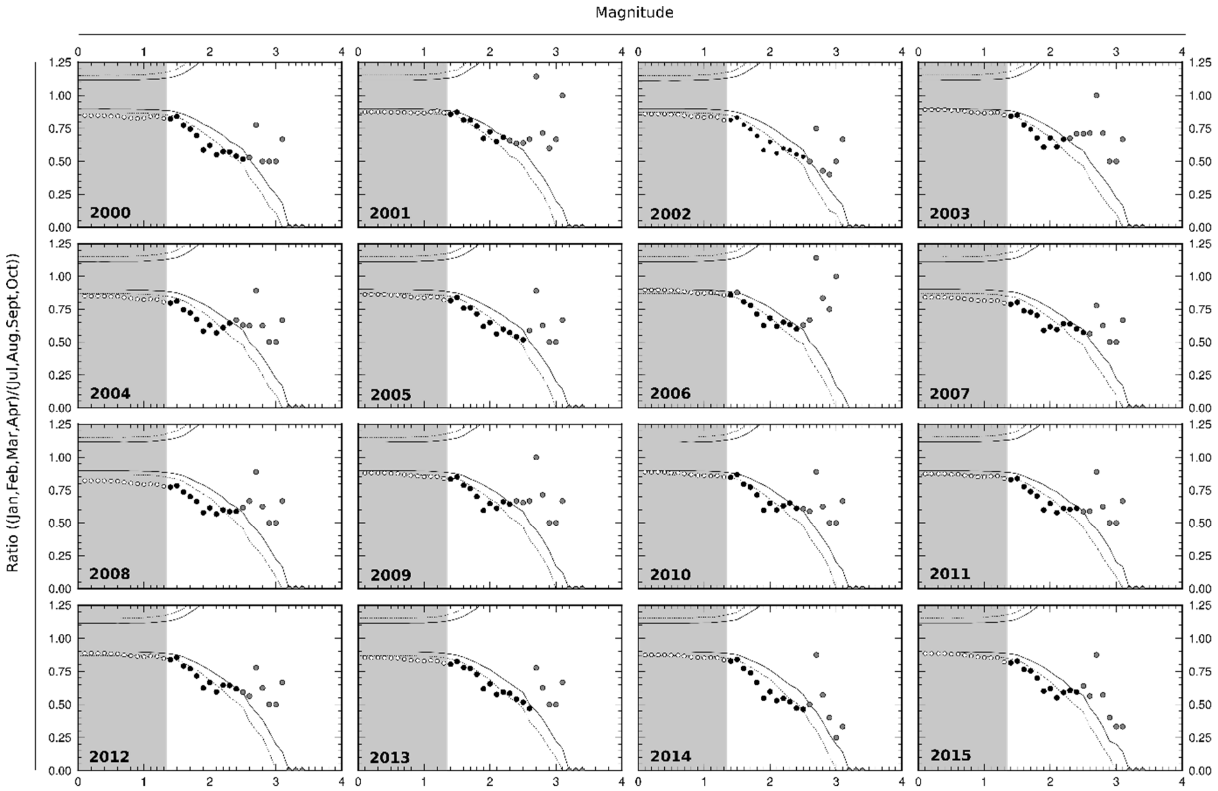

Supplementary Figure 4 | Full jack-knife analysis for complete seismic catalogue (Reelfoot fault region). Ratio of the number of earthquakes occurring in the four-month period encompassing January, February, March, April, to those occurring in July, August, September, October as a function of cut-off magnitude for the full seismic catalogue in the Reelfoot fault region (red box, Figure 1a). Grey shaded areas indicate the magnitude of completeness. Light and dark blue areas indicate the 99% and 95% confidence limit respectively. Black points are those where the ratio exceeds the 95% confidence limit. Number in the lower left corner of each panel indicates the year of data removed from the 1/1/2000 to 31/12/2015 overall time period.

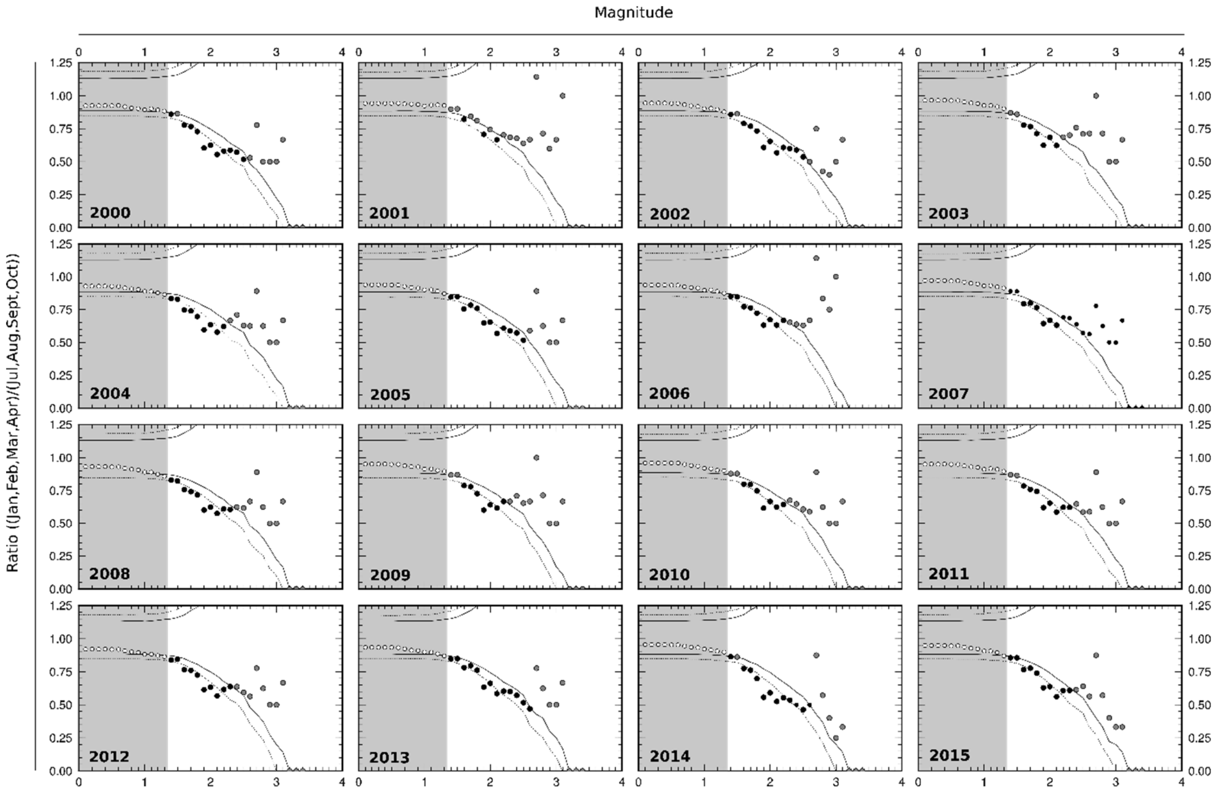

Supplementary Figure 5 | Full jack-knife analysis for declustered seismic catalogue (Reelfoot fault region). Ratio of the number of earthquakes occurring in the four-month period encompassing January, February, March, April, to those occurring in July, August, September, October as a function of cut-off magnitude for the declustered seismic catalogue in the Reelfoot fault region (red box, Figure 1a). Grey shaded areas indicate the magnitude of completeness. Light and dark blue areas indicate the 99% and 95% confidence limit respectively. Black points are those where the ratio exceeds the 95% confidence limit. Number in the lower left corner of each panel indicates the year of data removed from the 1/1/2000 to 31/12/2015 overall time period.

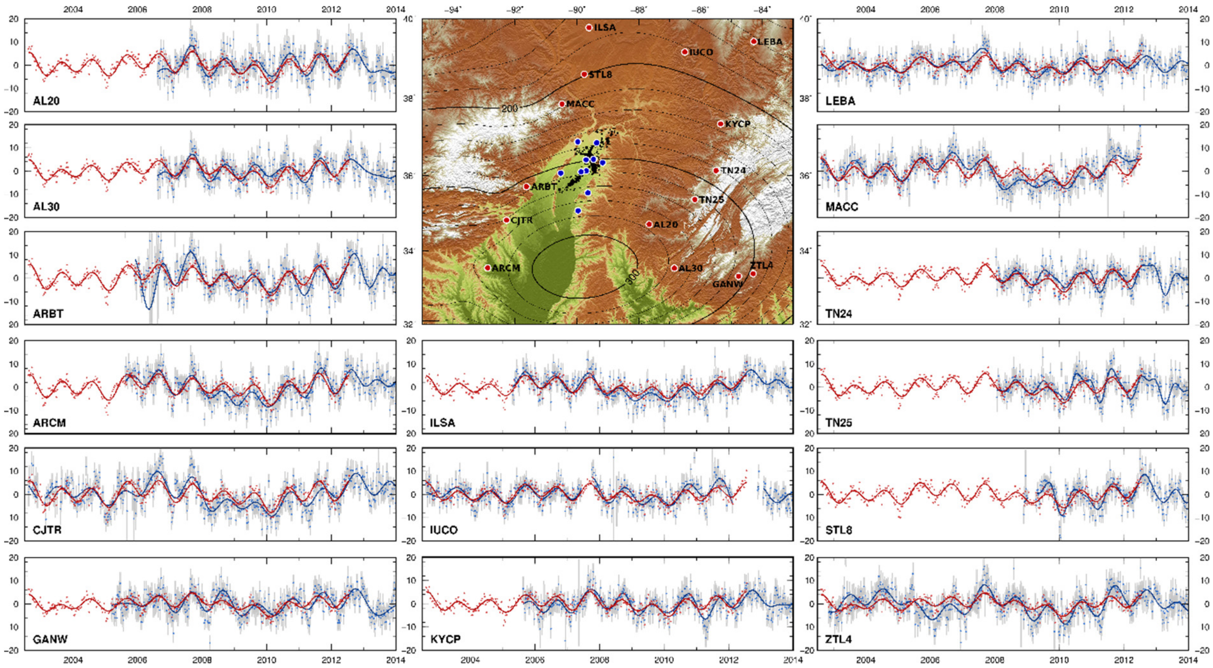

Supplementary Figure 6 | GPS displacement observations and gravity-derived displacement predictions across the wider CEUS. Vertical-component GPS displacements for 15 sites distributed across the wider Central and Eastern US, For processing details, see Methods. Site locations are given in Supplementary Table 1. Timeseries are corrected for instrumentation-related offsets. Grey bars are 1σ uncertainties. GPS site locations, identified by the site ID shown in the lower left of each panel are shown on the map. Blue points are the locations of the timeseries shown in Figure 3. Map contours are for peak-to-peak annual variation in GRACE-derived gravity, expressed as Equivalent Water Height in millimetres. Red points on the timeseries are vertical-component displacements calculated for a visco-elastic spherical Earth response to GRACE-observed surface load variations (see Methods). A best-fit linear trend has been removed from both GPS and gravity-derived timeseries. Blue and red lines on timeseries panels show a best fit 24-component fourier series to the GPS and gravity timeseries respectively.

GPS Site	*Latitude (°)*	*Longitude (°)*	*Observations period (yrs)*	*Data Completeness*	*Stacked Seasonal Amplitude[3] (mm)*	*Mean Annual Amplitude[4] (mm)*
ARPG[1]	36.06	-90.52	8.98	99%	9.4	11.9
BLMM[1]	36.88	-89.97	11.52	99%	10.3	12.9
CVMS[1]	35.54	-89.64	12.73	81%	7.7	14.3
HCES[1]	36.33	-89.17	13.95	85%	10.4	14.3
MAIR[1]	36.85	-89.36	14.43	78%	7.4	10.7
MCTY[1]	36.12	-89.70	14.28	83%	8.2	11.6
NWCC[1]	36.42	-89.46	14.05	74%	12.5	14.6
PTGV[1]	36.41	-89.70	14.53	87%	8.1	10.9
STLE[1]	36.09	-89.86	14.26	79%	7.5	11.0
ZME1[1]	35.07	-89.96	11.39	94%	8.9	11.9
AL20[2]	34.71	-87.66	7.98	99%	8.0	10.9
AL30[2]	33.53	-86.85	7.98	99%	7.4	10.0
ARBT[2]	35.71	-91.63	8.98	93%	9.7	15.6
ARCM[2]	33.54	-92.88	8.98	98%	7.3	10.1
CJTR[2]	34.82	-92.27	14.58	91%	7.6	11.2
GANW[2]	33.31	-84.77	9.40	97%	6.0	9.4
ILSA[2]	39.78	-89.61	9.28	97%	5.6	8.3
IUCO[2]	39.17	-86.51	14.23	86%	6.3	8.6
KYCP[2]	37.34	-85.35	8.96	94%	6.4	9.2
LEBA[2]	39.43	-84.28	12.63	99%	5.6	8.2
MACC[2]	37.85	-90.48	14.58	80%	6.3	8.9
STL8[2]	38.61	-89.76	5.69	90%	9.2	10.8
TN24[2]	36.13	-85.50	6.56	98%	7.4	9.5
TN25[2]	35.37	-96.19	6.51	99%	8.2	10.1
ZTL4[2]	33.38	-84.30	11.74	94%	6.8	10.4

Supplementary Table 1 | GPS Data Table. GPS site locations, observation epochs, percentage completeness of the timeseries over that epoch (taken from Craig and Calais[29]), and amplitude of the annual variation. Those sites labelled [1] are those from Figures 1 & 3. Those labelled [2] are those from Supplementary Figure 9. [3]Note that the stacked seasonal amplitude underestimates the true amplitude of the short-term variations, due to the maximum-amplitude peaks not exactly coinciding in each year. [4]Mean annual amplitude is calculated by taking the average amplitude of the annual variation within a 1-year period around each observation in the timeseries.
